# Financing of adolescent health and wellbeing in Bangladesh, Ethiopia and Nigeria: a case study of the Global Financing Facility

**DOI:** 10.1186/s44263-026-00332-4

**Published:** 2026-09-22

**Authors:** Olivia Biermann, Emilia Lindquist, Khadija Mitu, Adesola Olumide, Oloruntomiwa Oyetunde, Workneh Yadete, Sara Bylund, Sara Luckenbill, Mariam Claeson, Sarah Baird

**Affiliations:** 1 Department of Global Public Health, Karolinska Institutet, Tomtebodavägen 18a, Stockholm, 17177 Sweden; 2 Department of Anthropology, University of Chittagong, Hathazari, Chattogram, 4331 Bangladesh; 3 Institute of Child Health, College of Medicine, University of Ibadan, Queen Elizabeth Road, Agodi, Ibadan, Oyo State Nigeria; 4 Quest Research, Training and Consultancy Public Limited Company, Woreda 11, Nifas Silk Lafto Sub, Addis Ababa, Ethiopia; 5 Department of Global Health, Milken Institute School of Public Health, George Washington University, 950 New Hampshire Avenue NW, Washington, DC USA

**Keywords:** Adolescent health and well-being, Financing, Global Financing Facility for Women, Children and Adolescents, Case study, Bangladesh, Ethiopia, Nigeria

## Abstract

**Background:**

Adolescent health and wellbeing (AHW) remains largely underfunded with uneven progress across countries. This study investigates the Global Financing Facility for Women, Children, and Adolescents (GFF) in-country approach to AHW financing – including investment case prioritization, multisectoral stakeholder platforms, program implementation, monitoring and evaluation, and co-financing – examining what these processes have achieved for AHW, and the barriers limiting their impact, across Bangladesh, Ethiopia, and Nigeria.

**Methods:**

This collective case study triangulates country data from document reviews, key-informant interviews with multisectoral stakeholders, focus group discussions with adolescents, teachers and healthcare workers, and a global review of GFF investment cases. Transcripts were analysed using content analysis. Document data were extracted in Excel, then triangulated with the qualitative data. Reporting follows the COREQ checklist.

**Results:**

We identified five themes: (1) Adolescents are less prioritised than other age groups in country investment cases, with the limited attention to adolescents typically directed at female reproductive health. (2) The GFF uses pre-existing multi-stakeholder platforms in Bangladesh, Ethiopia, and Nigeria with limited involvement of youth. (3) The GFF supports implementation of AHW through the health sector-wide approach in Bangladesh, while engagement of the education sector is limited. In Ethiopia, adolescent-friendly health services are included in the national financial plan, but implementation faces sociocultural barriers such as perceived stigmatisation and lack of privacy. Nigeria has a national adolescent-specific implementation plan, but a lack of coordination between federal and state levels hinders progress. (4) Monitoring and evaluation (M&E) is weak, despite agreement on adolescent health indicators. Only Nigeria has an adolescent M&E framework. (5) The GFF supports co-financing through donor alignment. Domestic resources are key to progress, although the actual amount of the budget allocation and impact on AHW specific outcomes remain unknown.

**Conclusions:**

The GFF co-financing model has increased attention to AHW and aligned investors, but progress is insufficient to drive transformative change. The GFF can be optimized by overcoming coordination and measurement challenges, increasing application of innovative financing instruments for AHW, and meaningfully engaging youth. Given the reduction in development financing globally, countries can draw useful lessons from the GFF model to reduce the gap in adolescent health funding.

**Supplementary Information:**

The online version contains supplementary material available at https://doi.org/10.1186/s44263-026-00332-4.

## Background

Globally, the current generation of 10-24-year-olds faces growing challenges to their health and wellbeing, with increased financing and targeted actions needed to improve the trajectory of adolescent health and wellbeing (AHW) outcomes [1]. This is particularly important given the ‘triple dividend’ from investment in AHW, whereby investing in adolescents will not only shape their present and future lives, but also that of the next generation [1, 2]. In addition, adolescence is also a critical period for the onset of many health conditions, particularly mental health disorders, which can have long-lasting consequences throughout the life course [3]. Adolescence is also a critical window for establishing health behaviours and preferences—including diet, physical activity, and substance use—that shape the risk of non-communicable diseases across the life course [1]. New evidence is also emerging on the return on investment in AHW, with modelling studies suggesting that preventing and treating adolescent mental disorders and suicide can generate significant returns through improved productivity, reduced healthcare costs, and enhanced lifetime outcomes [4–6]. While AHW is increasingly mentioned in national and global policies and strategies, such as the Sustainable Development Goals and the Global Strategy for Women’s, Children’s, and Adolescents’ Health, these agendas remain largely underfunded [7, 8].

Across countries, the level of financing towards AHW is insufficient, uneven, and is disproportionate to the magnitude of challenges that adolescents face [9]. For example, development assistance for adolescent health cumulatively accounted for only 1.6% of development health assistance from 2003 to 2015, increasing to only 2.4% from 2016 to 2021, even though adolescents make up close to 25% of the global population [1, 10]. This underinvestment is also reflected in research efforts on adolescent health, which in turn influences priority-setting and policy development [11]. Increased financial investments across sectors are needed to match the disease burden of adolescents, paying attention to demographic and epidemiological trends including the growing burden of mental illness and obesity. Investments must draw on human rights, social, health, and economic perspectives [1, 12].

The Global Financing Facility for Women, Children, and Adolescents (GFF) is a country-led partnership for Reproductive, Maternal, Newborn, Child, and Adolescent Health and Nutrition (RMNCAH-N) set up in 2015 to close the financial gap to end preventable maternal and child deaths and improve adolescent health by 2030. The GFF is in more than 36 countries as of 2026 [13]. Hosted by the World Bank and governed by the Investors Group, the GFF uses trust funds to mobilize additional domestic resources, link with concessional financing, and to connect with private capital markets and other partners. At the core of the GFF country approach is the development of a country-led investment case, developed by the government in collaboration with development partners, civil society, the private sector, and youth representatives, often through a country platform for RMNCAH-N. The investment case serves as a prioritization tool through which GFF partners aim to strengthen coordination, reduce fragmentation, and enhance the impact of investments. The focus on adolescent health has increased in the 2021–2025 and 2026–2030 GFF strategy periods [14, 15].

Previous studies have reviewed the inclusion of adolescent sexual and reproductive health in GFF country documents and highlight that while adolescents are recognised as a focus group, their presence in investment cases and other key program implementation and monitoring documents is weak and varied, with limited financial resources and a lack of standardized monitoring indicators as top reasons for the lack of attention [16, 17]. We generated preliminary findings from this work for the *second Lancet Commission on adolescent health and wellbeing* and summarized as key findings in panel 16 and the appendix [1]. The full analysis is reported here for the first time, with a discussion of the implications for the evolving financial landscape, with reductions and unpredictability in development financing for AHW and across maternal, newborn, child, and adolescent health, and nutrition [1].

This study aims to investigate the GFF’s in-country approach to AHW financing—including investment case prioritization, multisectoral stakeholder platforms, program implementation, monitoring and evaluation, and co-financing—examining what these processes have achieved for AHW, and the barriers limiting their impact, across Bangladesh, Ethiopia, and Nigeria. It draws on learnings from these three countries, situated within available evidence from a global review of country investment cases.

## Methods

This is a collective case study, examining multiple cases to gain a comprehensive understanding of the extent and ways by which the GFF has contributed to increased attention, financial resources, and allocation of these resources for program implementation of interventions to improve AHW outcomes [18]. The methods are briefly described below, while Supplementary Material 1: Detailed information on methods provides all details for the global review as well as each step in the qualitative data collection and analysis per country, using the COnsolidated criteria for REporting Qualitative research (COREQ) checklist [19] (Supplementary Material 2: COREQ Checklist).

The three country case studies were selected from a diverse set of countries enrolled in the GFF. Each of the three countries was chosen due to the existence of specific programs targeting adolescents through diverse approaches: Bangladesh, with a component on multisector engagement, specifically including schools; Ethiopia, one of the first GFF countries, with an explicit focus on adolescent-friendly health services in the national health sector plan; and Nigeria, addressing AHW through a decentralized state-level nutrition program [20–22]. The findings from the three country studies were complemented by a global review of investment cases of 30 of the 36 GFF countries to have a benchmark for GFF investment cases across all enrolled countries; the remaining six countries had no investment case publicly available at the time of extraction. Investment cases were retrieved from the GFF data portal [23], and the full list of documents reviewed is provided in Supplementary Material 1: Detailed information on methods.

To identify documents relevant to AHW in the three countries, local GFF liaison officers, government officials, and other interviewees shared key documents. Documents were also identified through local websites of the GFF, the World Bank, government ministries, and international and local organizations. The documents were reviewed to understand how AHW is prioritized compared to other RMNCAH-N areas. The data sources included GFF investment cases and country materials, national policies and strategies, programme and implementation documents, World Bank financing and project reports, as well as analytical reviews and partner reports related to adolescent health and broader health system strengthening. In total, we reviewed 73 national documents, including 28 from Bangladesh, 22 from Ethiopia, and 23 from Nigeria. For more details see Supplementary Material 1: Detailed information on methods.

Purposive and snowball sampling were used to select in-country interviewees, aiming for the sample to comprise varied perspectives from multisectoral key-stakeholders with knowledge of the GFF and/or in-country investments in AHW, as well as current or former GFF representatives. Interviewees included government officials from ministries responsible for health, finance, education, and youth at both the national and sub-national levels; GFF liaison officers and GFF Secretariat staff; World Bank and other development partner representatives; implementing partners and technical agencies; and civil society and youth organisations engaged in the country platforms. Potential interviewees were identified in collaboration with the GFF Secretariat and the GFF country offices, adding individuals through snowball sampling. Purposive and snowball sampling was also used for the in-country focus group discussions (FGDs) with beneficiaries and implementers, including adolescents and young people (aged 15–28) interested in discussing their health and wellbeing, as well as teachers and healthcare workers knowledgeable on the topic.

AO, KM, OO, and WY recruited the participants and conducted the semi-structured key-informant interviews and FGDs in Ethiopia (12 interviews and 5 FGDs with 28 participants between August–October 2023), Bangladesh (11 interviews and 5 FGDs with 40 participants between February–May 2024), and Nigeria (10 interviews and 6 FGDs with 58 participants between August–November 2024). Table 1 provides an overview of the data sources.

The interview guides included questions about opportunities and challenges for investing in AHW, programme activities and multisectoral collaboration, youth engagement, and the GFF’s investments in and impact on AHW. The FGD guides focused on adolescents’ experiences and needs related to their health and wellbeing, as well as opportunities for youth engagement. As an example, the topic guide from the case study in Bangladesh is available in Supplementary Material 3: Topic guides (Bangladesh). Others are available on request. Interviews and FGDs were voice-recorded using mobile phones or Zoom (version 6.0.0; Zoom Communications, Inc., San Jose, CA, USA) and transcribed, and then translated into English, where applicable.

EL and OB analyzed the qualitative data from interviews and FGDs in close collaboration with AO, KM, OO, and WY, using content analysis [24] in the software Taguette [25]. Most codes were pre-defined based on the GFF logic model, while new codes were added to the codebook throughout the analysis process [23]. For more information see Supplementary Material 1: Detailed information on methods. The GFF logic model is structured across six stages: inputs, thematic areas, activities (planned/implemented), outputs, outcomes, and impact.

SBy, SL, and EL extracted relevant information from the global and national documents into Microsoft Excel (version 16.112.3; Microsoft Corporation, Redmond, WA, USA). The data extraction indicators included: (i) having a dedicated section on adolescent health and wellbeing (AHW), (ii) AHW mentioned in relation to specific health and education topics, (iii) AHW mentioned in relation to other multisector collaborations, (iv) stakeholders mentioned, (v) multi-stakeholder platform mentioned, (vi) geographical focus (if yes, geographical focus mentioned for AHW), (vii) marginalized/vulnerable adolescents mentioned, (viii) specific objectives related to AHW, (ix) initiatives related to AHW, (x) implementation plan including targets and indicators related to AHW, (xi) specific goal related to indicators, (xii) monitoring and evaluation plan related to AHW, (xiii) cost estimates related to AHW, (xiv) cost estimates for the document/plan, (xv) general information on the funding landscape, and (xvi) specific funders mentioned. The data from the document reviews were then triangulated with the qualitative data.

**Table 1:** Overview of the data sources

	Bangladesh	Ethiopia	Nigeria	Global review
Focus group discussions	*n = 5*	*n = 5*	*n = 6*	*n/a*
Key-informant interviews	*n* = 11	*n* = 12	*n* = 10	n/a
Investment cases	*n* = 1	*n* = 1	*n* = 1	*n* = 30
National documents	*n* = 28	*n* = 22	*n* = 23	n/a

Abbreviations: n; number of data sources; n/a; not applicable

## Results

Across Bangladesh, Ethiopia, and Nigeria, the GFF has contributed to increased attention to AHW: adolescent health priorities are incorporated into national investment cases, policies, and strategic plans in all three countries, and GFF-supported multisectoral stakeholder platforms are active in each setting. The GFF’s co-financing approach has helped align donors around shared AHW goals, and disbursement-linked indicators (DLIs) tied to adolescent health milestones have created financial accountability mechanisms. At the same time, progress across the GFF’s key in-country processes remains uneven and constrained by implementation barriers, as detailed in the sections below.

The findings across the country cases, and where relevant globally, are amalgamated under the following sub-headings corresponding to the GFF in-country process: (1) the GFF investment cases, (2) the GFF multisectoral stakeholder country platforms, (3) program implementation, (4) monitoring and evaluation (M&E) indicators, mechanisms, and structures, and (5) co-financing and use of innovative financing instruments. Table 2 summarises the AHW policy and implementation context in Bangladesh, Ethiopia, and Nigeria under GFF engagement. It is worth noting that national policies and strategies listed reflect the AHW policy landscape in each country; several were established prior to GFF engagement and represent the enabling environment for GFF investment, rather than direct outputs of GFF implementation. Indicators in the M&E row are drawn from national government strategies and frameworks. DLIs linked to World Bank Group financing are presented separately in Table 3. 

**Table 2:** Summary of the AHW policy and implementation context in Bangladesh, Ethiopia, and Nigeria under GFF engagement

	Bangladesh	Ethiopia	Nigeria
AHW included in the investment case/ in national planning documents	Yes, in the Health, Nutrition and Population Strategic Investment Plan 2016–2021. AHW priorities include SRH and nutrition.	Yes, in the Health Sector Transforma-tion Plan II 2020/21–2024/25. Priority areas for AHW include access to adolescent-friendly health services and SRH.	Yes, in the Investment case 2017–2030; Reproductive, Maternal, Newborn, Child, Adolescent health and nutrition. AHW priority areas include access to adolescent-friendly health services and information and SRH.
AHW specific national policy and strategy documents and guidelines	Costed National Action Plan for Adolescent Health Strategy 2020–2030	Adolescent and Youth Engagement Guidelines 2018–2025	Adolescents and Young People Implementation Plan 2021–2025
	National Adolescent Strategy 2017–2030	National Adolescents and Youth Health Strategy 2021–2025	National Monitoring and Evaluation Framework for Adolescent and Young People’s Health in Nigeria for 2021–2025
	National Plan of Action for Adolescent Health Strategy 2017–2030		National Policy on the Health and Development of Adolescents and Young People 2020–2024
	National Strategy for Adolescent Health 2017–2030		National Youth Policy 2019–2023
AHW costed or budgeted	The Costed National Action Plan for Adolescent Health Strategy 2020–2030 dedicates BDT 28,127 million (USD 232 million) 30% to SRH, 29% to nutrition, 16% to mental health	The National Adolescents and Youth Health Strategy 2021- 2025 commits ETB 2,755,539,628.32 (USD 49.2 million) to adolescent health	The Investment Case 2017–2030 allocates 3% of total RMNCAH-N funding (USD 50 million) to piloting adolescent- friendly health services
Multisectoral stakeholder country platform	*Development Partner Coordination Platform* established to implement the investment case but no information available regarding adolescent engagement	*Joint Core Coordinating Committee* is composed of members from the Health, Population and Nutrition Group. The *Joint Consultative Forum* is the technical arm. No details on adolescent involvement	Country platform established but no details regarding involvement of stakeholders including adolescents
Implementation progress and selected highlights	Implementation progress on AHW across sectors hindered by weak multisector collaboration despite financial incentives to engage education sector, and geographical and socio-economic disparities	AHW prioritized but gap between priorities and implementation progress; weak multisector collaboration, and sociocultural barriers to access to adolescent- friendly services	Nutrition schemes and adolescent girls’ education program implemented to increase access, but poor multisector collaboration, and lack of accountability between levels of the federal system
Monitoring and evaluation strategies and indicators	The National Plan of Action for Adolescent Health Strategy 2017–2030: indicators related to SRH, violence, nutrition, and mental health among adolescents	The National Adolescent and Youth Health Strategy lists 56 indicators related to AHW, some specific to boys	The National Monitoring and Evaluation Framework for Adolescent and Young People’s Health 2021- 2025: 19 core and 15 recommended adolescent specific indicators
Co-financing mechanisms	The GFF contributes to alignment of donors through the sector-wide approach (SWAp) and supports pooled funding of priorities in the Health, Nutrition and Population Strategic Investment Plan	Multidonor financing pooled through the Sustainable Development Goals Perfor-mance Fund, aiming to close financial gaps of the Health Sector Transformation Plan II	The GFF supports mobilization of domestic resources and collaboration with non- state actors to strengthen the uptake of community nutrition services among adolescent girls in particular

### The GFF investment cases

To provide a global benchmark for interpreting the three-country findings, the review of GFF investment cases in 30 countries shows that adolescents are less prioritised than other age groups in the country investment cases, compared with mothers, newborns and children under five, and that AHW is predominantly mentioned in relation to female sexual and reproductive health (SRH). Other topics, such as adolescent mental health, are mentioned only briefly, if at all, with no targeted interventions or indicators. Only four of the investment cases identify vulnerable adolescent populations, such as adolescents living in rural communities or with disabilities. While most investment cases (23 out of 30) highlight the need to invest in adolescent-friendly health services, M&E indicators to measure implementation progress are sparse.

Considering the 13 investment cases that provide a full breakdown of funding, investments in AHW average 7% (0.15–28%) of total funding for RMNCAH-N, which is considerably lower than average expenditure for maternal and newborn health (21%), child health (14%, predominantly directed toward children under five years of age), and nutrition (10%), although not mutually exclusive. Mauritania (28%), Zambia (20%), and Burkina Faso (8%) allocate the greatest proportion of their RMNCAH-N budget to AHW [26–28]. Based on the three countries that have updated their investment cases from 2024 onwards, the directional shift in AHW investments at country level varies, dropping in Kenya (5% in 2016 to 0.5% in 2025) [29], remaining stable in Madagascar (3.9% in 2020 to 4.1% in 2025) [30] and increasing in the Central African Republic (2.3% in 2020 to 15.7% in 2024) [31]. The investment cases do not specify the involvement of adolescents in the development process.

In Bangladesh, Ethiopia, and Nigeria, the amount and proportion of funding dedicated to AHW is not consistently reported across investment cases. Bangladesh’s investment case (2016–2021) totals USD 15,008,000,000, with explicit allocation for RMNCAH-N or AHW. The World Bank committed USD 1,195,000,000 and the GFF USD 25,000,000, with USD 1,026,000,000 and USD 2,900,000 disbursed respectively in 2023–2024 [32, 33]. Ethiopia’s investment case (2021–2025) dedicated 7.8% (USD 130,878,000) of total investment case funds towards RMNCAH-N issues without specifying the share for AHW. The World Bank and GFF allocated USD 400,000,000 of which USD 104,980,000 had been disbursed by 2025. The GFF share alone was USD 45,000,000 [34, 35]. Nigeria’s investment case (2017–2030) allocated 6.7% (USD 10,000,000) directly to AHW, alongside USD 225,000,000 committed through the World Bank and USD 7,000,000 through the GFF [36].

Investment cases are intended to identify priorities and mobilize and align financing from government, the GFF, the World Bank, and other partners toward those priorities. Evidence from the three countries is mixed on whether this objective were achieved. In Ethiopia, qualitative findings suggest that GFF involvement sparked broader investments in RMNCAH-N and helped align financing flows among funders. In Bangladesh, co-financing through the SWAp supported donor coordination, though concerns persist about value for money and the sustainability of external financing. In Nigeria, domestic financing remains limited, with most AHW investment continuing to flow from external sources and only a few states committing budget lines to adolescent health. Across all three countries, the amounts actually disbursed for AHW, and attributable to the GFF investment cases, cannot be assessed or validated due to limited financial information, including lack of disaggregation in plans and budgets, and financialreports on AHW spending – a gap that future public expenditure reviews could address.

Attention to AHW has increased in recent years, and governments have invested more in AHW in the last decade. Despite this, gaps persist between priorities outlined in the three investment cases and the level of current investments, which focus mainly on SRH. Other prioritized AHW areas identified in key documents, such as access to adolescent services (all countries), gender-based violence (Bangladesh), and promoting health behavior (Ethiopia) do not receive commensurate attention or funding. Although adolescents were mentioned in the development of most investment cases globally, including the three countries in this study, their involvement and active engagement could not be validated through the qualitative data.

### GFF multisectoral stakeholder country platforms

The GFF uses existing multisectoral stakeholder platforms for health in all three countries, designed to convene government ministries, development partners, UN agencies, civil society organizations (CSOs), the private sector, and youth. Findings suggest that CSO and youth involvement has been limited across the three countries. The engagement of other actors, including development partners and the private sector, was not systematically captured through qualitative data collection and represents a limitation of this analysis.

In Bangladesh, qualitative findings indicate poor understanding regarding roles and responsibilities of the multi-stakeholder platform. Only two interviewees had knowledge of the stakeholder platform, with one stating that it has “*played a pivotal role in the socio-economic development of Bangladesh*” while another interviewee commented that “*the platform is no longer available*”.

In Ethiopia, the members of a multi-stakeholder platform meet regularly, but the involvement of civil society organizations and youth remain limited. While one interviewee stated that the GFF has contributed to strengthening the country platform, another interviewee noted that there is “*no clear understanding about the GFF*,* especially on the Joint Core Coordinating Committee (multi-stakeholder platform). [...] There is no functional platform*”. The qualitative data indicates that engagement of adolescents and CSOs in the platform remains limited, though intended by the government [37, 38].

In Nigeria, qualitative findings confirm the existence of a multi-stakeholder platform that is co-chaired by the Ministries of Health and Social Welfare, and Women Affairs and Social Development. Adolescents participate in the platform through civil society, but the extent of their involvement is unknown. One interviewee suggests that the platform is currently active at the national level with ongoing efforts “*to work with states on how to replicate something like this at state level*”.

### Program implementation

In Bangladesh (2016), Ethiopia (2021), and Nigeria (2017), GFF investment cases were endorsed and incorporated into national policy and strategic documents for AHW (Table 2). Progress since then has been uneven across the three countries. GFF financing modalities, including DLIs (Table 3) and co-financing arrangements, have incentivized some government commitments, but structural barriers including weak multisectoral coordination and limited domestic financing have constrained implementation. The actual implementation progress of national policies and strategies remains a challenge, mainly due to lack of collaboration across sectors that share responsibility for AHW and weak monitoring and evaluation (discussed below).

In Bangladesh, poor collaboration and strategic planning, as well as geographical and socio-economic disparities, hinder effective implementation of AHW investments, according to key informants. Despite “*good things happening at the policy level*,* translation into action is more difficult*”. Interviewees indicate poor collaboration between sectors, notably, between the Ministries of Health and Education, impeding implementation of multisector AHW initiatives: “*The minute you get any type of political coordination as a condition; it is very difficult. Politicians find it very hard to coordinate with one another*”, noted a key-informant. Challenges to implementation are further exacerbated by tight budgets to tackle geographical and demographic disparities, as well as large school dropouts caused by the COVID-19 pandemic, which, at the time, made it more difficult to reach the adolescent population. The GFF supports the implementation of Bangladesh’s investment case and key health objectives through the SWAp, i.e., by assisting the government to organize partner funds and improve connections between interrelated areas [20]. The World Bank published two reports in 2024, confirming the implementation of an adolescent girls’ program in two of the country’s eight divisions (Chittagong and Sylhet), and a school-based health and nutrition program that had the potential to reduce girls’ dropout rates [39, 40]. The latter is reflected in a DLI. However, as noted by key informants, this is a missed opportunity since “*the Ministry of Education did not execute the program and did not spend the USD 10 million*”.

In Ethiopia, implementation efforts related to AHW remained insufficient according to interviews and FGDs. The Health Sector Transformation Plan II does not include an adolescent-specific implementation strategy [38]. Moreover, a gap persisted between initiatives outlined in the plan, such as adolescent nutrition and promoting healthy behaviours, and practice. For example, adolescents emphasized that “*school feeding programs have been interrupted due to supply shortages*” and that there are “*lots of psychologically affected students*” who do not get the support they need. Implementation efforts have also been hindered by disagreements between the three ministries responsible for AHW. For example, the implementation of a sexual health course in schools, supported by the United Nations Children’s Fund (UNICEF), was hindered due to disagreements between the Ministries of Health and Education regarding cultural implications of the content. The government and partners have made efforts to increase access to adolescent-friendly health services, a goal related to one DLI, but according to key informants, many adolescents remain reluctant to seek care due to the fear of facing stigma, disrespect, and lack of privacy.

Nigeria has developed an extensive set of policy frameworks and strategies focusing exclusively on AHW but has fallen short on implementation due to poor coordination. Interviewees stated that “*Nigeria has done very well in terms of developing appropriate policies*” however “*there is no strategy on how to achieve the policy goals*”. As policy in Nigeria is developed at the federal level and then contextualized to state needs, implementation is often fragmented and uncoordinated. In particular, stakeholders of the State fail to be responsive and accountable, “*it is now the role or the responsibility of donor organizations to hold the government accountable to ensure that donor funds are used for their purpose*”. Key-informants emphasized the importance of actively involving states throughout federal policy development to enhance implementation efforts.

**Table 3:** Disbursement Linked Indicators related to AHW in GFF countries

Examples of DLIs related to adolescents	Country	Timeframe	Status (March 2026)
Improved grade retention and cycle completion (Adolescent Girls Program)	Bangladesh	Dec 2017 - Jun 2023 (original end date: Dec 2022)	Revised target fully achieved [32].
School-based adolescent Health and Nutrition Program is developed and implemented	Bangladesh	Jul 2017 - Jun 2024 (original end date: Dec 2022)	The number of districts where the school-based adolescent health program met the target [33].
Percentage of health centers with youth-friendly health services	Ethiopia	Dec 2022- Jun 2026	As of Oct 2025, no data available for this indicator as the SARA/SPA survey has not been conducted as planned [34].
Development and endorsement of key primary health care unit guidelines and procedures that include adolescents and youth	Ethiopia	Dec 2022- Jun 2026	The indicator partially achieved the result in November 2024, and disbursement was made in May 2025 [35].
Annual increase in the number of adolescents utilizing health services at the Sub-District and community levels	Ghana	Jun 2023 - Jun 2026	As of Nov 2024, good progress has been made towards the achievement of the end targets for Adolescent health [41].
Percentage of secondary schools offering sexual and reproductive health services	Mozambique	April 2018 - Dec 2023	Target was restructured in 2022. Restructured target was met [42].
Evidence of new knowledge for nutrition and adolescent health results	Nigeria	Jun 2018 - Dec 2024 (original end date: Dec 2023)	Following restructuring, this DLI became known as a Performance Based Condition. Achievement unclear [36].

### M&E mechanisms, structures and indicators

There is agreement on M&E indicators in all three countries, particularly related to adolescent pregnancy. Disbursement Linked Indicators (Table 3) are associated with achievement of results and milestones linked to World Bank funding, offering opportunities for scaled financing and programmatic impact. However, the lack of functioning M&E systems hampers the measurement of progress towards those targets.

In Bangladesh, there is no M&E system that routinely monitors adolescent-specific indicators. As such, information on progress of indicators is not available. The qualitative data indicates that M&E is “*one of the weakest areas*” and “*there has always been a lack of monitoring and evaluation of the programs*”.

In Ethiopia, adolescent-specific indicators exist, predominantly related to improving the quality of adolescent-friendly health services and SRH, but systematic M&E to routinely measure and report on progress, is still lacking. One interviewee noted that an M&E “*strategy by itself is not enough. There should be stakeholders who are responsible to regularly monitor achievements [related to AHW]”.*

Nigeria has strategies and platforms for adolescent-specific M&E, but historically poor implementation hinders its success. One interviewee described M&E as one of “*the weakest links in the Nigerian adolescent health sector. The provision for monitoring is generally poor. The attention to monitoring is generally poor. Even when it is done*,* it is not done properly and is often not talked about*”. In particular, the qualitative data highlights a lack of nationally representative disaggregated data for adolescents, meaning there are no baselines against which to measure progress, hindering evaluation. To address this gap, Nigeria launched a National Youth Index, which “*looks at (adolescent) health*,* wellbeing*,* education*,* employment*,* and even access to finance*”. Interviewees believe the Index launched in August 2024, but that it is not yet integrated into wider, national monitoring tools.

### Co-financing

Globally, the GFF promotes the use of innovative financing instruments, such as Program-for-Results in fragile governance environments (which are common in GFF countries), Investment Project Financing (Nigeria), and DLIs. The latter are indicators agreed on between Ministries of Finance and the World Bank to trigger disbursement of funds on achievement of agreed milestones or results. The GFF also uses other financing instruments to make progress on country goals, such as social impact investment (where investors aim to generate both financial return and positive, measurable social or environmental impact) for newborn survival (Cameroon), and buy-down of interest on World Bank loans against achievement of health and nutrition outcomes (Guatemala). These opportunities are yet to be specifically applied to achievement of adolescent health outcomes. 

In the three country case studies, innovative financing instruments have contributed to thealignment and commitment of government and donors around AHW. While key-informant interviews report increased resources for AHW, particularly in the last decade, the amounts cannot be assessed or validated due to limited financial information in plans, budget documents, and national implementation reports. Further economic analysis, including public expenditure reviews, would be required.

In Bangladesh, the qualitative data suggests a need to get more value for money since “*if thegovernment’s own funding is not utilised properly, the technical funding provided by the GFF (and other donors) cannot and will not be utilised properly*”. There is also a worry that “*when Bangladesh graduates from the United Nations least developed country status by 2026, funding from international donors will reduce*”, which may challenge the ability to obtain sustainable, long-term funding commitments. This is particularly troublesome for AHW, as currently “*adolescents are somewhat overlooked” and “the funding landscape for family planning and adolescent health is already on a reducing trend*” according to key-informants.

In Ethiopia, the GFF’s involvement has sparked investments in RMNCAH-N by a range offunders and helped align financing flows. Details on amounts of investments specifically for AHW remain unclear and ongoing conflict within the country may make it challenging to continue receiving external health funding and implementing sustainable health initiatives. One interviewee emphasizes a “*need to better integrate NGO [non-governmental organization] and private sector funding – this will avoid redundant activities, as resources and activities can be allocated more appropriately, and this will also create transparency*”. Meanwhile, details on investments in AHW remain unclear since “*there is no specific adolescent health and wellbeing related funding mechanism in the current health system andthere is no budget[line] allocated for the purpose of improving adolescent health*”.

In Nigeria, the qualitative data indicates that “*political will and domestic funding, even at thestate level, is very low and minimal, so, you see many [health] interventions, at national and state level, come from external sources*”. This is reflected in AHW investments with “*only a few states committing budget lines to adolescent health and wellbeing*”. Key-informants emphasize that “if Nigeria is not willing to provide the necessary [domestic] funding to match up with what GFF is investing, it will be a challenge to achieve the goals”.

## Discussion

This case study examined how AHW is financed through the GFF’s in-country processes in Bangladesh, Ethiopia, and Nigeria, drawing on a review of 73 national documents, 33 key-informant interviews, and 16 focus group discussions, benchmarked against a global review of 30 GFF investment cases. We found that AHW is increasingly reflected in national investment cases, policies and strategic plans, and that multisectoral country platforms and AHW-related disbursement-linked indicators have created entry points for attention and accountability. However, adolescents remain less prioritised than other RMNCAH-N groups, with discussions around AHW predominantly framed around female SRH. M&E for AHW remains is limited, and committed resources do not consistently translate into implemented and monitored interventions.

The second Lancet Commission on adolescent health and wellbeing predicted that without major investments in adolescent SRH, education, and nutrition, a larger share of adolescents will suffer from increased morbidity and premature mortality by the end of the Sustainable Development Goals 2030 compared to the start in 2015 [1]. This remains unlikely given that most low- and middle-income countries face serious financial constraints, including competition between and within sectors for domestic resources and unpredictable donor funding in an uncertain global aid and development context.

The findings from Bangladesh, Ethiopia, and Nigeria are broadly consistent with patterns observed across the 30 GFF investment cases reviewed globally: adolescents receive less attention than other RMNCAH groups, AHW is predominantly framed around female SRH, and M&E indicators for AHW remain sparse. The average 7% AHW share of RMNCAH-N funding across the 13 investment cases with full funding breakdowns reflects a structural under-prioritisation that the three country cases confirm and extend. Importantly, the three-country findings reveal that implementation challenges compound this underfunding: even where AHW is included in investment cases and national plans, poor intersectoral coordination, weak M&E systems, and limited domestic resource commitments constrain progress. This suggests that the challenge for the GFF extends beyond raising AHW’s share of investment case funding, to ensuring that committed resources translate into implemented and monitored interventions at country level. This pattern is not confined to our three countries. A layered policy analysis of GFF investment cases and World Bank project appraisal documents across 28 countries reached a similar conclusion: although the GFF supported priority-setting and learning, translating priorities into resourced actions proved challenging, and accountability gaps and limited civil society engagement constrained country ownership [43]. Earlier content analyses of adolescent SRH in GFF planning documents likewise found adolescents recognised rhetorically but weakly represented in investment cases and monitoring documents [16, 17]. Our findings go further by showing that the gap persists beyond the planning stage through the implementation and monitoring and evaluation processes.

Based on the findings of this study, adolescents are increasingly included in national investment cases, policies, and plans but AHW must also be made explicit in the costing of interventions across RMNCAH-N to inform allocation of resources towards AHW outcomes, and in government budgets. In Bangladesh, Ethiopia, and Nigeria, AHW is articulated in national plans and strategies, yet budget allocations remain inconsistent: Bangladesh’s IC does not specify an AHW allocation, Ethiopia dedicates 7.8% of IC funds to RMNCAH-N without disaggregating AHW, and Nigeria, while allocating 6.7% directly to AHW, faces significant gaps between planned and actual spending. As has been seen in the mobilization of resources towards child health, the backbones of any successful national child survival program have been the focus on service coverage indicators, which track progress towards a core set of child health outcomes, sub-nationally, nationally, and globally [44]. A similar accountability approach is needed for AHW. The country strategies and implementation plans that prioritise adolescents provide opportunities to expand the scope of AHW from a focus on female SRH to include other AHW priorities, such as nutrition, mental health, and education. Bangladesh’s Costed National Action Plan for Adolescent Health 2020–2030 is a notable example, allocating 30% to nutrition and 16% to mental health alongside SRH, demonstrating that a broader scope is feasible when priorities are explicitly costed. There are also opportunities to increase the focus on adolescent boys. These findings also bear on the wider debate about what global health initiatives should catalyse. It has been argued that the GFF has not yet fully realised its potential to shift countries towards sustainable, domestically financed health investment in line with the Lusaka Agenda [45, 46]. Our three country cases illustrate that in the absence of AHW-specific budget lines and expenditure tracking, neither the extent of domestic commitment to adolescents nor its effect on AHW outcomes can be demonstrated.

Multisectoral stakeholder platforms are central to the GFF model, designed to convene government ministries, development partners, UN agencies, CSOs, the private sector, and youth to coordinate health investment across sectors. Findings from the three countries suggest these platforms have had mixed effectiveness, with coordination challenges and limited CSO and youth engagement common across contexts. The extent to which this engagement is “adequate” depends on the standard applied: frameworks such as Hart’s ladder of participation offer a spectrum from tokenistic inclusion to genuine co-decision-making [47], and by this measure, engagement across the three countries – where adolescents participate primarily through civil society rather than directly – sits toward the lower end. Analyses of GFF processes in other settings report the same pattern. A critical review of the GFF’s first phase concluded that meaningful adolescent and young people’s engagement in planning processes is nearly absent, and that adolescent priorities lose visibility as plans move from policy through to budget [48]. A case study of decision-making in Tanzania similarly showed how power asymmetries within country platforms shape whose priorities are reflected in investment decisions [49]. That the same dynamic appears across Bangladesh, Ethiopia, and Nigeria suggests it is structural to the platform model rather than specific to any one country. The GFF could strengthen its platform model by establishing explicit minimum standards for youth and civil society engagement, consistent with WHO guidance on meaningful adolescent participation in health programming [50].

We also learned that innovative financing instruments applied explicitly to AHW across sectors offer a window of opportunity for government commitment to increase sustainable financing for AHW and improve country outcomes for adolescents and the broader community. The DLIs related to achievement of AHW milestones illustrate both increased government financial commitments and accountability for results and milestones related to adolescents. In Bangladesh, DLIs for grade retention among adolescent girls and school-based health programs demonstrate cross-sectoral application of performance-based financing. In Ethiopia, a DLI on youth-friendly health services has partially achieved its target. In Nigeria, however, the DLI on adolescent health knowledge was restructured and its achievement remains unclear, reflecting the variation in DLI effectiveness across country contexts. This variation is consistent with the broader evidence on results-based financing in low- and middle-income countries, where systematic review evidence points to gains in service utilisation and coverage but an evidence base that remains partial and inconclusive, with few evaluations addressing cost-effectiveness or sustainability [51]. The same caution applies when such instruments are extended to AHW: the value of AHW-related DLIs will depend on whether their achievement is transparently monitored and reported, which was not consistently the case in our three countries.

To gain full momentum for AHW, systematic and transparent monitoring of progress on AHW outcomes and other key indicators are needed within countries and globally. A set of indicators has been suggested by the second Lancet Commission on adolescent health and wellbeing, and the Global Action for Measurement of Adolescent Health (GAMA) initiative [1, 52]. The GFF has been a consistent member of the GAMA working group since its inception, being involved in proposing and advocating for AHW indicators. The importance of tracking a core set of indicators has been seen in “exemplar” countries that have made progress on maternal and child survival. For example, M&E has played a key role in achieving measurable progress towards child mortality reduction in Bangladesh and maternal mortality reduction in Ethiopia, which could also be applied to adolescent health [53]. If nationally implemented, Nigeria’s National Youth Index could drive progress and serve as a good example of comprehensive M&E with attention to AHW that could be adapted in other countries.

Several strengths and limitations should be considered when interpreting these findings. The strengths of this collective case study are the focus on in-country experiences, the use of various data sources and a common codebook, systematic and interactive data analysis, and the triangulation of findings. Contextual insights, however, are limited by the selection of participants who were mostly based in the capital cities. Moreover, the lack of access to government financial data limits insights into actual budget allocation, disbursements, and spending of funds for AHW across sectors, geographically, and at decentralised levels. That said, in many contexts data disaggregated for adolescents simply does not exist.

## Conclusions

The GFF has contributed to an increase of attention and financial resources for AHW in Bangladesh, Ethiopia, and Nigeria, mainly through alignment of donors, but progress has not been enough to drive transformative change for adolescents. The GFF’s co-financing model can be optimized by overcoming coordination and measurement challenges, capitalizing on the use of innovative financing instruments, and meaningfully engaging youth and civil society organizations. Lessons from these three countries are particularly timely given the launch of the 2026–2030 strategy [15], which seeks to deepen country-led approaches and expand the GFF’s reach to 50 countries. The renewed commitment of donors, reflected in the over USD 800 million pledged at the 2026 replenishment launch, signals continued international support for expansion of this model [46, 54]. At the same time, given the reduction and unpredictability of global development financing, the GFF’s country-driven approach offers useful lessons to reduce the financial gaps across reproductive, maternal, newborn, child, and adolescent health and nutrition.

## Supplementary Information

Below is the link to the electronic supplementary material.

Supplementary Material 1

Supplementary Material 2

Supplementary Material 3

## Data Availability

The datasets generated and analysed during the current study are not publicly available due to ethics approvals requiring us to protect the confidentiality of our participants. Through full transcriptions, participants may be identifiable. Anonymized data segments may be available from the corresponding author (olivia.biermann@ki.se) on reasonable request, subject to ethical approval conditions.

## Abbreviations

AHW: Adolescent health and well–being
COREQ: Consolidated criteria for reporting qualitative research
DLI: Disbursement–linked indicator
FGD: Focus group discussion
GAMA: Global Action for Measurement of Adolescent Health
GFF: Global Financing Facility for Women, Children, and Adolescents
IC: Investment case
M&E: Monitoring & Evaluation
RMNCAH: N–Reproductive, Maternal, Newborn, Child, and Adolescent Health and Nutrition
SWAP: Sector–wide approach
SRH: Sexual and reproductive health
