# Supplementary material for: Financing of adolescent health and wellbeing in Bangladesh, Ethiopia and Nigeria: a case study of the Global Financing Facility
Source: BMC Glob Public Health. 2026 Sep 22;4:93. doi: 10.1186/s44263-026-00332-4 (PMC13599230; doi:10.1186/s44263-026-00332-4)
Supplement: Supplementary file 1 [file 44263_2026_332_MOESM1_ESM.docx]

**Financing of adolescent health and wellbeing in Bangladesh, Ethiopia and Nigeria: a case study of the Global Financing Facility**

**Supplementary Material 1. Detailed information on methods**

**Overview of the data sources**

|  | **Bangladesh** | **Ethiopia** | **Nigeria** | **Global review** |
| --- | --- | --- | --- | --- |
| **Focus group discussions** | n=5 | n=5 | n=6 | n/a |
| **Key-informant interviews** | n=11 | n=12 | n=10 | n/a |
| **Investment cases** | n=1 | n=1 | n=1 | n=30 |
| **National documents** | n=28 | n=22 | n=23 | n/a |

**Codebook used for qualitative analysis based on GFF Logic Model**

| **Inputs** | **Thematic areas** | **Activities (planned/**  **implemented)** | **Outputs** | **Outcomes** | **Impact** |
| --- | --- | --- | --- | --- | --- |
| Stakeholder engagement | Gender equality | Country platform | National investments aligned | Improvements in AHW service delivery | Accelerated improvements in indicators |
| Available data & evidence | SRHR | Convening global investors | Donor investments aligned | Financing reform adopted and implemented | Strengthened platform for PHC/UHC |
| Available financing | MNCH | Convening national investors | National and donor investments aligned | Systems reform adopted and implemented |  |
| Available technical assistance | Nutrition | Investment Case | AHW prioritized in financing reform | Coverage of high impact interventions |  |
| Available global investments | Education | Financing reforms identified | AHW prioritized in systems reform | Increased/sustained resources |  |
| Attention to AHW* | Essential services | Systems reforms identified | Disbursement* | Improved efficiency |  |
|  | Mental health* | Adolescent engagement* | Budgeting* | Improved health system performance* |  |
|  | Immunization* | NGO/CSO engagement* |  | Lack of AHW services |  |
|  | Violence* | AHW strategy developed* |  | Transparency* |  |
|  | Drugs and alcohol* | Multisector collaboration* |  | Challenges* |  |
|  | Lack of trust in health services* |  |  | Increased/sustained stigmatization* |  |
|  |  |  |  | Removed stigmatization* |  |

*Additional codes added that are not included in the GFF logic model

Abbreviations used in the table: AHW=adolescent health and wellbeing; SRHR=sexual and reproductive health and rights; MNCH=maternal, newborn and child health; NGO/CSO=non-governmental organization/civil society organization; PHC/UHC=primary health care/Universal Health Coverage

**Bangladesh country case study**
*Document review*

Search and data extraction process

Documents were collected by searching the Global Financing Facility for Women, Children, and Adolescents (GFF), and World Bank websites for “Bangladesh” from October 2023 to March 2024, and by asking the GFF liaison officer to suggest additional documents. Of the twenty-eight documents included, three were from the GFF, thirteen from the World Bank, eight from Bangladesh’s Ministry of Health and Family Welfare (MoHFW), one from Ministry of Education (MoE), one from Ministry of Women and Children Affairs (MoWCA), and two from multilateral organizations and non-governmental organizations, respectively. Documents included from the World Bank and GFF consisted of financial agreements, project/program appraisal documents, and status reports, while the MoHFW, MoE, and MoWCA documents mainly consisted of strategic plans, action plans, and guidelines.

Relevant information was extracted from the documents into Excel. The data extraction indicators included: dedicated section on adolescent health and wellbeing (AHW), AHW mentioned in relation to specific health and education topics, AHW mentioned in relation to other multisector collaboration, stakeholders mentioned, multi-stakeholder platform mentioned, geographical focus (if yes, geographical focus mentioned for AHW), marginalized/vulnerable adolescents mentioned, specific objectives related to AHW, initiatives related to AHW, implementation plan including targets and indicators related to AHW, specific goal related to indicators, monitoring and evaluation plan related to AHW, cost estimates related to AHW, cost estimates to the document/plan, general information on the funding landscape, and specific funders mentioned.

Included documents:

1. Global Financing Facility for Women, Children, and Adolescents Bangladesh. Bangladesh: Closing Existing Gaps while Tackling New Challenges. Dhaka; GFF; 2023.
2. Global Financing Facility for Women, Children, and Adolescents. GFF Country Spotlight: Bangladesh. Dhaka: GFF; 2018.
3. Ministry of Education Bangladesh. Adolescent Student Program. Dhaka: Ministry of Education Bangladesh; 2018.
4. Ministry of Health and Family Welfare Bangladesh. Adolescent Health Training Manual (for teachers and health service providers); Adolescent Health Programme. Dhaka: Ministry of Health and Family Welfare Bangladesh; 2018.
5. Ministry of Health and Family Welfare Bangladesh. Costed National Action Plan for Adolescent Health Strategy 2020-2030. Dhaka: Ministry of Health and Family Welfare Bangladesh; 2020.
6. Ministry of Health and Family Welfare Bangladesh. Expanding Social Protection for Health Towards Universal Coverage; Health Care Financing Strategy 2012-2032. Dhaka: Ministry of Health and Family Welfare Bangladesh; 2012.
7. Ministry of Health and Family Welfare Bangladesh. Health Sector Support Project (HSSP). Including Additional Financing Updated Social Management Framework. Dhaka: Ministry of Health and Family Welfare Bangladesh; 2017.
8. Ministry of Health and Family Welfare Bangladesh. Health, Nutrition and Population Strategic Investment Plan 2016–2021: Better Health for a Prosperous Society. Dhaka: Ministry of Health and Family Welfare Bangladesh; 2016.
9. Ministry of Health and Family Welfare Bangladesh. National Plan of Action for Adolescent Health Strategy 2017-2030. Dhaka: Ministry of Health and Family Welfare Bangladesh; 2016.
10. Ministry of Health and Family Welfare Bangladesh. National Strategy for Adolescent Health 2017-2030. Dhaka: Ministry of Health and Family Welfare Bangladesh; 2016.
11. Ministry of Health and Family Welfare Bangladesh. Second National Plan of Action for Nutrition (2016-2025). Dhaka: Ministry of Health and Family Welfare Bangladesh; 2017.
12. Ministry of Women and Children Affairs Bangladesh. Bangladesh National Adolescent Strategy. Dhaka: Ministry of Women and Children Affairs; 2020.
13. Sultan M, Nazneen S. Policy and Legal Analysis Notes: Bangladesh. A Review of the National Strategy for Adolescent Health. Dhaka: Gender & Adolescence: Global Evidence (GAGE): 2018.
14. United Nations Fund for Population Activities. Investing in Young People for a Better Future. Dhaka: UNFPA; 2020.
15. World Bank. Adolescent Health for Education and Health Sectors Program: School-based Adolescent Health Nutrition and Population Program. Dhaka: World Bank; 2018a.
16. World Bank. Global Financing Facility Grant Agreement (Health Sector Support Project) between the People's Republic of Bangladesh and International Development Association; Acting as Administrator of the Multi-Donor Trust Fund for the Global Financing Facility in Support of Every Woman Every Child. Dhaka: World Bank; 2017.
17. World Bank. Implementation Completion and Results Report on a Program in the Amount of US$ 874.9 million to the People's Republic of Bangladesh for the Bangladesh - Health Sector Development Program (P118708) December 5, 2017. Dhaka: World Bank; 2017.
18. World Bank. Implementation Completion Report Review. Health Sector Development Program. Dhaka: World Bank; 2018.
19. World Bank. Implementation Status and Results Report. Health Sector Support Project. Dhaka: World Bank; 2023.
20. World Bank. Implementation Status and Results Report. Health Sector Support Project. Dhaka: World Bank; 2024.
21. World Bank. International Development Association: Project Appraisal Document on a Proposed Credit in the Amount of SDR 149 Million (US$200 Equivalent) to the People's Republic of Bangladesh for an Urban Health, Nutrition and Population Project. August 9, 2023. Dhaka: World Bank; 2023.
22. World Bank. International Development Association: Implementation Completion and Results Report on a Credit in the Amount of SDR 366.6 Million (US$510 Million Equivalent) and a Grant from the Global Financing Facility in the Amount of US $10 million to the People's Republic of Bangladesh for the Transforming Secondary Education for Results Operation. January 26, 2024. Dhaka: World Bank; 2024.
23. World Bank. International Development Association: Program Appraisal Document on a Proposed Credit in the Amount SDR 366.6 Million (US$510 Million Equivalent) and a Grant in the Amount of US$10 million to the People's Republic of Bangladesh for a Transforming Secondary Education for Results Operation November 23, 2017. Dhaka: World Bank; 2017.
24. World Bank. International Development Association: Project Appraisal Document on a Proposed Credit in the Amount of SDR361.2 Million (US$500 Million Equivalent) and a Grant in the Amount of US$15 Million to the People's Republic of Bangladesh for a Bangladesh Health Sector Support Project July 7, 2017. Dhaka: World Bank; 2017.
25. World Bank. International Development Association: Project Appraisal Document on a Proposed Grant in the Amount of SDR 109.3 Million (US$150 Million Equivalent) of which SDR 91.083 Million (US$125 Million Equivalent) is from the IDA 18 Regional Sub-window for Refugees and Host communities to the People's Republic of Bangladesh for a Health and Gender Support Project for Cox's Bazar District. Dhaka: World Bank; 2020.
26. World Bank. International Development Association: Project Appraisal Document on a Proposed Credit in the Amount of SDR 222.8 Million (US$300 Million equivalent) to the People's Republic of Bangladesh for an Economic Acceleration and Resilience for NEET (EARN). June 06, 2023. Dhaka: World Bank; 2023.
27. World Bank. International Development Association: Restructuring Paper on a Proposed Program Restructuring of Transforming Secondary Education for Results Operation: Approved on December 18, 2017 to the People's Republic of Bangladesh. Dhaka: World Bank; 2023.
28. World Bank. The Primary Health Care System of Bangladesh; A Primary Health Care Performance Initiative Assessment June 2022. Dhaka: World Bank; 2022.

*Qualitative data collection* *and analysis*

Purposive and snowball sampling was applied to select interviewees in Bangladesh, aiming for the sample to comprise multisectoral key-stakeholders with knowledge of the GFF and/or investing in AHW in-country. Potential interviewees were identified in collaboration with the GFF Secretariat and the GFF country office, while also adding individuals by snowballing throughout the data collection process. Purposive and snowball sampling was also used for the focus group discussions (FGDs) with adolescents (aged 18-24) interested in discussing their health and wellbeing, as well as the FGDs with teachers interested in the topic.

The guides for interviews and FGDs were contextualized to Bangladesh, obtaining feedback from the GFF liaison officers and using information from the document review. The interview guides included questions about opportunities and challenges for investing in AHW, programme activities and multisectoral collaboration, youth engagement, and the GFF’s investments in and impact on AHW. The FGD guides focused on adolescents’ experiences and needs related to their health and wellbeing, as well as opportunities for youth engagement. The full guides are available upon request.

KM (female), a professor and experienced qualitative researcher, and her four research assistants collected data between 1 February and 31 May 2024 in Chittagong and Dhaka. KM had received verbal approval to proceed by the Chittagong University Ethics Approval Board on 1 February 2024, while the approval letter only arrived on 8 May 2024 due to administrative challenges at the Ethics Approval Board at the time. Four interviews were conducted before the arrival of the approval letter. Sixteen potential interviewees were contacted via e-mail or telephone; of those, three suggested colleagues to be interviewed instead, while none declined participation, one did not respond, and one cancelled last minute. After obtaining written informed consent, KM interviewed thirteen key-informants. Due to faulty recordings and interruptions, two interviews were not included in the final study. The remaining eleven key-informant interviews included representatives from the Bangladesh Ministry of Health and Family Welfare (n=2), World Bank (n=2), Ministry of Education (n=1), GFF Country Office (n=1), GFF Secretariat (n=1), World Bank Education (n=1), UNFPA (n=1), USAID (n=1), and academic institutions (n=1). Six interviewees were female, five were male. Interview location was based on the respondents’ preferences; five interviews took place in the respondents’ offices, five were conducted via Zoom, and one respondent sent responses in writing via email. KM did not conduct any repeat interviews. The average duration of the interviews was 50 minutes (35-72 minutes). During the interviews, no one besides KM and the respective respondent were present.

For the FGDs, KM contacted organizations that work with youth activists in Dhaka and selected one organization that works with improving youth SRH issues. For the school-based FGDs, KM contacted the principals of two secondary level schools in Chittagong: one all-girls school and one all-boys school. To recruit FGD participants, snowball sampling was used. KM contacted the youth activists in Dhaka via telephone, explaining the research objectives. Participants were recruited based on their willingness to participate. For the school-based interviews, research assistants contacted the students and their parents via telephone to obtain verbal informed consent, which was documented by KM. KM conducted five FGDs, comprising of two with teachers (n=11, female=4, male=7), one with youth activists (n=4) aged 18–21 years, one with female students (n=7), and one with male students (n=6). Students were aged 15–17 years old. The average duration of the FGDs was 60 minutes (45-98 minutes). During the FGD with the youth activists, only KM and the participants were present. During the school-based FGDs, four research assistants were involved under the supervision of KM. Two male researchers conducted the FGDs with male students and their teachers and two female researchers conducted the FGDs with female students and their teachers.

KM voice-recorded and transcribed the interviews and FGDs, which had been conducted in Bengali, and translated them into English. The transcripts were not returned to participants for comments. KM also conducted field notes, which were verbally shared with the team of co-authors for preliminary insights. EL and OB analyzed the qualitative data, in close collaboration with KM, using content analysis and employing the open-source qualitative analysis tool *Taguette*.^29^ Most codes were pre-defined based on the GFF logic model, while new codes were identified and added to the codebook throughout the analysis process.^30^ A summary of the findings was created, which was merged with data from the document review, and data across countries in the Findings section of the manuscript. The data was determined to have sufficient information power to reach the study’s aim.^31^

**Ethiopia country case study**
*Document review*

Search and data extraction process

Documents, identified via email from key-informants and by searching the GFF and World Bank websites for “Ethiopia”, were collected from August 2023 to January 2024. Of the twenty-two included documents, eleven were from the World Bank, seven from the Ministry of Health (MoH), and four from the GFF. The included World Bank and GFF documents mainly consisted of financial agreements and project/program appraisal documents, while the MoH documents comprised strategic plans and guidelines. Pertinent information was extracted into Excel. Data extraction indicators were the same as described for Bangladesh above.

Included documents:

1. Global Financing Facility for Women, Children, and Adolescents. Ethiopia Health Private Sector Assessment. Addis Ababa: GFF; 2019.
2. Global Financing Facility for Women, Children, and Adolescents. Financing Agreement. Ethiopia Program for Results (Hybrid) for Strengthening Primary Health Care Services between Federal Democratic Republic of Ethiopia and International Development Association. Addis Ababa: GFF; 2023.
3. Global Financing Facility for Women, Children, and Adolescents. GFF Planning, Alignment and Coordination in Ethiopia. Addis Ababa: GFF; undated.
4. Global Financing Facility for Women, Children, and Adolescents. Governance Document for the Global Financing Facility in Support of Every Woman Every Child. Addis Ababa: GFF; 2018.
5. Global Financing Facility for Women, Children, and Adolescents. Grant Agreement. Additional Financing for Health Sustainable Development Goals Program-for-Results between Federal Democratic Republic of Ethiopia and International Bank for Reconstruction and Development IDA (acting as administrator of the GFF). Addis Ababa: GFF; 2017.
6. Ministry of Health Ethiopia. Adolescent and Youth Engagement Guideline 2018-2025. Addis Ababa: Ministry of Health Ethiopia; 2020.
7. Ministry of Health Ethiopia. Brief on Health Status of Adolescent and Youth Health in Ethiopia. Addis Ababa Ministry of Health Ethiopia; undated.
8. Ministry of Health Ethiopia. Health Harmonization Manual. Second Edition. Addis Ababa: Ministry of Health Ethiopia; 2020.
9. Ministry of Health Ethiopia. Health Sector Transformation Plan II. 2020/21-2024/25 (2013 EFY - 2017 EFY). Addis Ababa: Ministry of Health Ethiopia; 2021.
10. Ministry of Health Ethiopia. National Adolescents and Youth Health Strategy (2021-2025). Addis Ababa: Ministry of Health Ethiopia; 2021.
11. Ministry of Health Ethiopia. National Health Equity Strategic Plan 2020/21-2024/25. Addis Ababa: Ministry of Health Ethiopia; 2020.
12. Ministry of Health Ethiopia. Stakeholder Engagement Plan. PforR for Strengthening Primary Health Care Services Project. Addis Ababa: Ministry of Health Ethiopia; 2022.
13. World Bank. Appraisal Environmental and Social Review Summary Appraisal Stage (ESRS Appraisal Stage). Ethiopia Program for Results (Hybrid) for Strengthening Primary Health Care Services. Addis Ababa: World Bank; 2022.
14. World Bank. Environmental and Social Commitment Plan (ESCP). Ethiopia Program for Results for Strengthening Primary Health Care Services. Addis Ababa: World Bank; 2022.
15. World Bank. Ethiopia Health Sustainable Development Goals Program for Results Additional Financing. Environmental and Social System Assessment (ESSA) Update. Addis Ababa: Ethiopia; 2017.
16. World Bank. Ethiopia. Program for Results for Strengthening Primary Health Care Services Technical Assessment. Addis Ababa: World Bank; 2022.
17. World Bank. Fiduciary Systems Assessment. Ethiopia Program for Results (Hybrid) for Strengthening Primary Health Care Services. Addis Ababa: World Bank; 2022.
18. World Bank. Implementation Status & Results Report. Ethiopia Program for Results (Hybrid) for Strengthening Primary Health Care Service. Addis Ababa: World Bank; 2023.
19. World Bank. Integrated Safeguards Data Sheet Concept Stage. Health MDGs P4R Additional Financing. Addis Ababa: World Bank; 2017.
20. World Bank. International Development Association: Program Appraisal Document on a Proposed Grant in the Amount of SDR 301.3 Million (US$400 Million Equivalent). A Proposed Grant in the Amount of US$25 Million from the Global Financing Facility Ethiopia. Second Round Grant. And a Proposed Grant in the Amount of US$20 Million from the Global Financing Facility Ethiopia Essential Health Services to the Federal Democratic Republic of Ethiopia for an Ethiopian Program for Results (Hybrid) for Strengthening Primary Health Care Services. Addis Ababa: World Bank; 2022.
21. World Bank. Program Information Documents. Ethiopia Program for Results (Hybrid) for Strengthening Primary Health Care Services. Addis Ababa: World Bank; 2022.
22. World Bank. Program paper on a proposed restructuring and a proposed additional financing in the amount of SDR 110.6 million (US$150 million equivalent) to the Federal Democratic Republic of Ethiopia with a proposed grant from the Global Financing Facility for Every Woman and Every Child in the amount of US$60 million and a proposed grant from the Power of Nutrition Trust Fund in the amount of US$20 million in support of the Health Sustainable Development Goals Program for Results. Addis Ababa: World Bank; 2017.

*Qualitative data collection and analysis*

The sampling process was the same as described for Bangladesh above. The guides for the interviews and focus group discussions (FGDs) included the same types of questions as in the examples mentioned above, with contextual adaptations and further adaptations after the first interview, which was considered a pilot interview. The full guides are available upon request.

WY (male), an experienced qualitative researcher, collected the data between 1 August and 31 October 2023 in Addis Ababa. Eighteen potential interviewees were contacted via e-mail or telephone: of those, three suggested colleagues to be interviewed instead, while two declined participation. After obtaining written informed consent from face-to-face interviewees and verbal consent from individuals interviewed via telephone (documented by WY), WY interviewed twelve key-informants, including representatives from the Ethiopia Ministry of Health (MoH) (n=3), Ministry of Finance (n=1), the GFF country office (n=1), the World Bank country office (n=1), funders (n=2), academic institutions (n=1), and civil society organizations (n=3). Nine interviewees were female, three were male. Four interviews took place in the respondents’ offices and eight were conducted via telephone, based on the respondents’ preferences. Apart from one interview which was conducted over the course of two days, WY did not conduct any repeat interviews. The average duration of the interviews was 40 minutes (17-64 minutes). During the interviews, no one besides WY and the respective respondent were present, except in one interview with a representative from the MoH where an advisor was present, but did not interfere in the interview.

For the FGDs, the GFF liaison office recommended WY to contact a civil society organization, implementing activities related to AHW. WY called the organization, which facilitated the contact to two local secondary schools in Addis Ababa that supported the recruitment of FGD participants. Selected students were beneficiaries of GFF funding through the work of local organisations, with demonstrated engagement in issues surrounding adolescent health and wellbeing. After obtaining written informed consent, WY conducted the FGDs, comprising participants from two local secondary schools, which were recommended by the GFF country office. Written informed consent for students under the age of 18 was provided by the school principal and/or school coordinator, as per local regulations. The five FGDs included one with male teaching staff aged 23–35 years (n=4), as well as two with male students (n=11 and n=8), and two with female students (n=8 and n=9) aged 17–20 years. The average duration of the FGDs was 60 minutes (49-102 minutes). During the FGDs, only WY, the participants, and a school coordinator were present.

WY voice-recorded and transcribed the interviews and FGDs, which he had conducted in Amharic language, and translated them into English. The transcripts were not returned to participants for comments. WY also conducted field notes, which he used to verbally share preliminary insights with the team of co-authors. EL and OB analyzed the qualitative data, in close collaboration with WY, using content analysis and employing the open-source qualitative analysis tool *Taguette*.^29^ Most codes were pre-defined based on the GFF logic model, while new codes were identified and added to the codebook throughout the analysis process (S3).^30^ A summary of the findings was created and merged with data from the document review, and data across countries in the Findings section of the manuscript. The data was determined to have sufficient information power to reach the study’s aim.^31^

**Nigeria country case study**

*Document review*

Search and data extraction process

Documents, identified via email from key-informants and by searching the GFF and World Bank websites for “Nigeria”, were collected from March to May 2024. Of the twenty-three included documents, ten were from the Ministry of Health, seven from the World Bank, two from the Ministry of Humanitarian Affairs, one from the Federal Government of Nigeria, one from the Ministry of Youth and Sports Development, one from the Ministry of Women and Gender Affairs, and one from the United Nations Population Fund. The included World Bank and GFF documents mainly consisted of financial agreements and project/program appraisal documents, while the ministerial documents comprised of strategic plans and guidelines. The UNFPA document is a country program implementation document. Pertinent information was extracted into Excel. Data extraction indicators were the same as described for Bangladesh above.

Included documents:

1. Federal Republic of Nigeria. National Monitoring and Evaluation of Adolescent and Young People’s Health in Nigeria. Monitoring and Evaluation Plan 2021 – 2025. Abuja: Federal Republic of Nigeria; 2021.
2. Federal Ministry of Health. Accelerating Nutrition Results in Nigeria; Project Implementation Manual. Abuja: Federal Ministry of Health; 2018.
3. Federal Ministry of Health. Adolescents and Young People Implementation Plan 2021 – 2025. Abuja: Federal Ministry of Health; 2020.
4. Federal Ministry of Health. Health Sector Recommendations for Nutrition Indicators collected nationally in Nigeria. Abuja: Federal Ministry of Health; 2022.
5. Federal Ministry of Health. Investment case 2017-2030; Reproductive, Maternal, Newborn, Child, Adolescent health and nutrition. Abuja: Federal Ministry of Health; 2017.
6. Federal Ministry of Health. National Child Health Policy. Abuja: Federal Ministry of Health; 2022.
7. Federal Ministry of Health. National Guidelines for the Prevention and Control of Micronutrient Deficiencies in Nigeria. Abuja: Federal Ministry of Health; 2021.
8. Federal Ministry of Health. National Policy on the Health and Development of Adolescents and Young People in Nigeria 2020 – 2024. Abuja: Federal Ministry of Health; 2020.
9. Federal Ministry of Health National Strategic Plan of Action for Nutrition 2021 – 2025. Abuja: Federal Ministry of Health; 2021.
10. Federal Ministry of Health. Nigeria Family Planning Blueprint 2020 – 2024. Abuja: Federal Ministry of Health; June 2020.
11. Federal Ministry of Health. National Social Protection Policy. Abuja: Federal Ministry of Humanitarian Affairs; 2016.
12. Federal Ministry of Health. Second National Strategic Health Development Plan 2018-2022. Abuja: Federal Ministry of Health; 2018.
13. Federal Ministry of Health. Strategic Roadmap 2021-2025. Abuja: Federal Ministry of Humanitarian Affairs; 2022.
14. Federal Ministry of Gender and Women Affairs. National Gender in Health Policy 2021 - 2026. Abuja: Federal Ministry of Gender and Women Affairs; 2021.
15. Federal Ministry of Youth and Sports Development. National Youth Policy 2019 – 2023. Abuja: Federal Ministry of Youth and Sports Development; 2019.
16. United Nations Fund for Population Activities. Country Programme Document for Nigeria 2023-2027. New York: UNFPA; 2022.
17. World Bank. Additional Financing for the Adolescent Girls Initiative for Learning and Empowerment Project between Federal Republic of Nigeria and International Development Association. Abuja: World Bank; 2023.
18. World Bank. Financing Agreement (Adolescent Girls Initiative for Learning and Empowerment Project) between Federal Republic of Nigeria and International Development Association. Abuja: World Bank; 2020.
19. World Bank. Implementation Status & Results Report. Adolescent Girls Initiative for Learning and Empowerment. Abuja: World Bank; 2023.
20. World Bank. Implementation Status & Results Report. Nigeria Accelerating Nutrition Results. Abuja: World Bank; 2023.
21. World Bank. International Development Association; Project Appraisal Document on a proposed credit in the amount of SDR 156.5 million (US$225 million equivalent) and a proposed grant in the amount of US$7 million from the Global Financing Facility to the Federal Republic of Nigeria for an accelerating nutrition results in Nigeria project; May 23, 2018. Abuja: World Bank; 2018.
22. World Bank. International Development Association; Project Appraisal Document on a proposed credit in the amount of SDR 364.5 million (US$500 million equivalent) to the Federal Republic of Nigeria for an Adolescent Girls Initiative for Learning and Empowerment Project; June 30, 2020. Abuja: World Bank; 2020.
23. World Bank. Project Paper on a proposed additional credit in the amount of SDR 88.2million (USD 125 million equivalent) and GFF grant in the amount of US20 million to the Federal Republic of Nigeria for the Nigeria State Health Investment Project. Abuja: World Bank; 2016.

*Qualitative data collection and analysis*

The guides for the interviews and focus group discussions (FGDs) included the same types of questions as in the examples mentioned above, with contextual adaptations and further adaptations after the first interview, which was considered a pilot interview. The full guides are available upon request.

AO (female), a professor and experienced qualitative researcher, led a team of six research staff (including a doctoral student) experienced in conducting qualitative research. The researchers collected the data between 1 August and 30 November 2024 in Kaduna state. Twenty potential interviewees were contacted via e-mail or telephone; of those, three suggested colleagues to be interviewed instead, while four declined participation, one did not respond, and two had to cancel the interview last minute. After obtaining written informed consent from face-to-face interviewees and verbal consent from individuals interviewed via Zoom (documented by AO), AO and her team interviewed ten key-informants, including representatives from the Nigerian Ministry of Health and Social Welfare (n=2), Ministry of Youth Development (n=2), GFF country office (n=1), United Nations Population Fund (n=1), academic institutions (n=1), and civil society organizations (n=3). Five interviewees were female and five were male. Seven interviews were conducted via Zoom, and three were conducted in person. One interview was conducted over two sessions (10 minutes and 42 minutes) due to poor internet connection. AO did not conduct any repeat interviews. The average duration of the interviews was 82 minutes (25-100 minutes). During the interviews, only participants were present.

For the FGDs, AO was directed to a Kaduna-based researcher with knowledge of the GFF. The recruitment of FGD participants occurred as follows: health workers were selected through the local government health authority, based on their involvement in the implementation of the ANRIN project. Teachers were recruited via their union, based on their familiarity with the AGILE project. For participants at the IDP camp in Angwan Zawu, the research team first consulted the camp leadership, who facilitated the identification of eligible participants. In-school adolescents were identified from their communities where the AGILE project had been implemented and based on their involvement.

After obtaining written informed consent, seven experienced research assistants located in Kaduna were recruited to conduct the FGDs due to nationwide protests preventing AO from travelling. The six FGDs included one with healthcare workers (n=10, female=9, male=1), one with teachers (n=10, female=2, male=8), one with male secondary school students (n=10), one with female secondary school students (n=8), and two with internally displaced people (n= 20, female=10, male=10). Students were aged 18-28 years old and internally displaced people were aged 18-25 years old. The average duration of the FGDs was 76 minutes (44-96 minutes). During the FGDs, only the interviewer, the participants, and a note-taker, who did not interfere with the interview, were present.

The researchers voice-recorded and transcribed the interviews and FGDs. The key-informant interviews were conducted in English and the FGD’s were conducted in Hausa language. Where relevant, transcripts were then translated into English. The whole process was supervised by AO. The transcripts were not returned to participants for comments. AO also consulted field notes, which were used to verbally share preliminary insights with the team of co-authors. EL analyzed the qualitative data, in close collaboration with AO and OO, using content analysis and employing the open-source qualitative analysis tool *Taguette*.^29^ Most codes were pre-defined based on the GFF logic model, while new codes were identified and added to the codebook throughout the analysis process (S3).^30^ A summary of the findings was created and merged with data from the document review, and data across countries in the Findings section of the manuscript. The data was determined to have sufficient information power to reach the study’s aim.^31^

**Global review**

*Document review (GFF investment cases)*

Search and data extraction process

A search on the GFF website was carried out in September 2023 to identify countries with investment cases. In total, thirty-one out of the thirty-six GFF countries had active investment cases, while five investment cases (Haiti, Madagascar, Myanmar, Pakistan, Tajikistan) were under development or awaiting approval. Of the thirty-one countries with active cases, three investment cases (Guinea, Indonesia, Vietnam) were not readily available on the GFF website. The GFF liaison officers from these three countries were contacted to enquire about their investment cases. The investment case from Guinea was obtained, thus twenty-nine investment cases were included in the study.

In June 2024 another search was conducted on the GFF website to follow up on the developing and absent investment cases. Indonesia’s and Vietnam’s investment cases were still unavailable. Two of the five investment cases under development had been approved (Madagascar, Pakistan). Only Madagascar’s investment case was uploaded on the GFF website and therefore added to the study. Pakistan’s GFF liaison officer was contacted, and an oral summary of the investment case was received via Zoom. No copy of the document was obtained and as such Pakistan was not included in the final study.

Data was extracted from the thirty included investment cases into Excel. Indicators for data extraction included: frequency of which RMNCAH-N terms were mentioned (“adolescent”, “maternal”, “child”, “newborn”, “nutrition”), health issues (adolescent pregnancy, maternal mortality, HIV, violence, alcohol and drug abuse, non-communicable diseases, mental health, nutrition), interventions (adolescent-friendly health services, school-based programs, community-based programs, adolescent engagement), adolescent-related objectives, gap analysis, stakeholders involved in producing the investment case, identification of vulnerable adolescent groups and priority regions, mention of multisectoral perspectives, as well as a break-down of RMNCAH-N funding, where available.

Included documents:

1. Afghanistan: Health Sector Thematic Working Group. [Internet]. Health Sector Transition Strategy 2023-2025. Afghanistan; 2022. [cited 2023 Sept 28]. Available from: <https://www.globalfinancingfacility.org/sites/default/files/Health%20Sector%20Transition%20Strategy%202023-2025.pdf>
2. Bangladesh: Ministry of Health and Family Welfare. [Internet]. Health, Nutrition and Population Strategic Investment Plan (HNPSIP) 2016-21. Bangladesh; 2016. [cited 2023 Sept 20]. Available from: <https://www.globalfinancingfacility.org/sites/gff_new/files/documents/Bangladesh-Investment-Case.pdf>
3. Burkina Faso: Ministere de la Sante. [Internet]. Dossier d'Investissment - Améliorer la Sante de la Reproduction, de la Mere, du Nouveau-Ne, de l’Enfant et de l’Adolescent-Jeune, de la Nutrition et de l’Etat Civil Et Statistiques Vitales. Burkina Faso; 2020. [cited 2023 Sept 15]. Available from: <https://www.globalfinancingfacility.org/sites/default/files/Burkina-Faso_Investment_Case_FR.pdf>
4. Cambodia: Ministry of Health. [Internet]. Overview of Cambodia’s Investment Case for RMNCAH-N. Cambodia; 2018. [cited 2023 Sept 10]. Available from: <https://www.globalfinancingfacility.org/sites/gff_new/files/Cambodia-GFF-Investment-Case.pdf>
5. Cameroon: République du Cameroun. [Internet]. Dossier D’investissement pour L’Amélioration de la Santé de la Reproduction, Sante de la Mère, du Nouveau-né, de L’Enfant et de L’Adolescent/Jeune au Cameroun 2017-2020. Cameroon; 2016. [cited 2023 Sept 5]. Available from: <https://www.globalfinancingfacility.org/sites/gff_new/files/documents/Cameroon_GFF_Investment_Case_Fr.pdf>
6. Central African Republic: Ministère de la Santé et de la Population. [Internet]. Dossier D’Investissement pour la Réduction de la Mortlité Maternelle, Néonatale et Infantojuvenile, et pour L’Amélioration de la Santé des Adolescents 2020-2022. Central African Republic; 2019. [cited 2023 Sept 3]. Available from: <https://www.globalfinancingfacility.org/sites/default/files/Central-African-Republic-Investment-Case-Fr.pdf>
7. Chad: Republique du Chad. [Internet]. Dossier D’Investissement Réduction De La Mortalité, Maternelle, Néonatale, Infantile, Infanto-Juvénile Et Amélioration de la Santé des Adolescents et de L’Enregistrement des faits D’état Civil di SRMNIAN-EC TCHAD 2023-2027. Chad; 2022. [cited 2023 Sept 5]. Available from: <https://www.globalfinancingfacility.org/sites/gff_new/files/documents/Republique-Tchad-Dossier-Investissement-GFF-SRMNIAN-2023-2027.pdf>
8. Congo: République Democratique du Congo. [Internet]. Plan National de Développement Sanitaire recadré pour la période 2019-2022: Vers la couverture sanitaire universelle. Democratic Republic of Congo; 2018. [cited 2023 Sept 2]. Available from: <https://www.globalfinancingfacility.org/sites/gff_new/files/documents/DRC_Investment_Case_FR.pdf>
9. Côte D’Ivoire: République du Côte D’Ivoire. [Internet]. Financement de la Santé Dossier D’Investissement 2020-2023. Côte D’Ivoire; 2019. [cited 2023 Sept 7]. Available from: <https://www.globalfinancingfacility.org/sites/gff_new/files/documents/Cote-dIvoire-Investment-Case_Resume-fr.pdf>
10. Ethiopia: Ministry of Health. [Internet]. Health Sector Transformation Plan II. 2020/21-2024/25 (2013 EFY - 2017 EFY). Ethiopia; 2021. [cited 2023 Sept 8]. Available from: <https://live-gff-drupal-9.pantheonsite.io/sites/default/files/Ethiopia-HSTP-II.pdf>
11. Ghana: Ministry of Health. [Internet]. Health Sector Medium Term Development Plan 2022-2025. Ghana; 2021. [cited 2023 Sept 18]. Available from: <https://www.globalfinancingfacility.org/sites/default/files/Ghana-GFF-Investment-Case.pdf>
12. Guatemala: Gobierno de la República de Guatemala. [Internet]. Cruzada Nacional por la Nutrición. Guatemala; 2018. [cited 2023 Sept 15]. Available from: <https://www.globalfinancingfacility.org/sites/default/files/2022-12/Guatemala-Investment-Case-Cruzada-Nacional-por-la-Nutricion_ES.pdf>
13. Guinea: Ministere de la Sante. [Internet]. Dossier d'Investissment pour la reduction de la mortalite maternelle, neonatale, infanto juvenile et des adolescents 2020-2024. Guinea; 2019. [cited 2023 Sept 23]. Available from: <https://www.globalfinancingfacility.org/sites/default/files/Guinea_GFF_Investment_Case_Fr.pdf>
14. Madagascar. Cadre d´investissement pour la santé reproductive, maternelle, néonatale, infanto-juvénile et de l`adolescent, et pour la nutrition à Madagascar 2020-2024. Madagascar; 2019. [cited 2024 June 28]. Available from: <https://www.globalfinancingfacility.org/sites/default/files/Madagascar%20Cadre%20d%27investissement%20SRMNIA-N_version%20finale%2001%20Dec%202021.pdf>
15. Kenya: Ministry of Health, Government of Kenya. [Internet]. Kenya Reproductive, Maternal, Newborn, Child and Adolescent Health (RMNCAH) Investment Framework. Kenya; 2016. [cited 2023 Sept 14]. Available from: <https://www.globalfinancingfacility.org/sites/gff_new/files/Kenya-Investment-Case.pdf>
16. Liberia: Ministry of Health of Republic of Liberia. [Internet]. Investment Case for Reproductive, Maternal, Newborn, Child, and Adolescent Health (2016-2020). Republic of Liberia; 2016. [cited 2023 Sept 26]. Available from: <https://www.globalfinancingfacility.org/sites/gff_new/files/Liberia-Investment-Case.pdf>
17. Mali: République du Mali. [Internet]. Dossier D’Investissement de la Santé de la Reproduction, de la Mere, du Nouveau-Ne, de L’Adolescent et de la Nutrition (SRMNEA+N 2019 -2023). Mali; 2021. [cited 2023 Sept 19]. Available from: <https://www.globalfinancingfacility.org/sites/default/files/2022-12/Dossier-investment-de-la-SRMNEA-N_Mali.pdf>
18. Malawi: Ministry of Health and Population Government of Malawi. [Internet]. The Government of Malawi’s Investment Case for Reproductive, Maternal, Newborn, Child and Adolescent Health and Nutrition 2020-2022. Malawi; 2019. [cited 2023 Sept 19]. Available from: <https://www.globalfinancingfacility.org/sites/gff_new/files/documents/Malawi-GFF-Investment-Case.pdf>
19. Mauritania: République Islamique de Mauritanie. [Internet]. Dossier d’investissement 2022-2025. Mauritania; 2022. [cited 2023 Sept 9]. Available from: <https://www.globalfinancingfacility.org/sites/gff_new/files/images/Mauritanie_Investment-Case-SRMNIA_fr.pdf>
20. Mozambique: Republic of Mozambique. [Internet]. Global Funding Mechanism in Support of All Women and All Children Investment Case Proposal - Version IV. Republic of Mozambique; 2017. [cited 2023 Sept 10]. Available from: <https://www.globalfinancingfacility.org/sites/default/files/Mozambique-Investment_Case-EN.pdf>
21. Niger: République du Niger. [Internet]. Dossier D’Investissement du Niger Pour la Santé Reproductive, Maternelle, Néonatale, Infantile, Adolescent, et la Nutrition (SRMNIA-Nut) 2022-2026. Niger; 2022. [cited 2023 Sept 17]. Available from: <https://www.globalfinancingfacility.org/sites/gff_new/files/documents/Niger-GFF-Investment-Case-FR.pdf>
22. Nigeria: Federal Republic of Nigeria. [Internet]. Investment Case 2017-2030 Reproductive, Maternal, Newborn, Child and Adolescent Health and Nutrition. Federal Republic of Nigeria; 2018. [cited 2023 Sept 5]. Available from: <https://www.globalfinancingfacility.org/sites/gff_new/files/documents/Nigeria-Investment-Case.pdf>
23. Rwanda: Government of Rwanda. [Internet]. National Early Childhood Development Program (NECDP) Strategic Plan 2018-2024. Rwanda; 2017. [cited 2023 Sept 6]. Available from: <https://www.globalfinancingfacility.org/sites/default/files/2022-12/Rwanda-Investment-Case.pdf>
24. Senegal: Ministère de la Santée et de L’Action Sociale. [Internet]. Réduction de la Mortalité Maternelle, Néonatale, Infanto-Juvénile, des Adolescents et des Jeunes. Senegal; 2019. [cited 2023 Sept 14]. Available from: <https://www.globalfinancingfacility.org/sites/gff_new/files/documents/Senegal-dossier-dinvestisement.pdf>
25. Sierra Leone: Ministry of Health and Sanitation. [Internet]. National Reproductive, Maternal, Newborn, Child and Adolescent Health Strategy 2017–2021. Sierra Leone; 2016. [cited 2023 Sept 25]. Available from: <https://www.globalfinancingfacility.org/sites/gff_new/files/documents/Sierra-Leone-GFF-Investment-Case.pdf>
26. Somalia: Ministry of Health of the Federal Government of Somalia. [Internet]. Investment Case for the Somali Health Sector 2022–2027. Somalia; 2021. [cited 2023 Sept 26]. Available from: <https://www.globalfinancingfacility.org/sites/default/files/Somalia-GFF-Investment-Case.pdf>
27. Tanzania: Ministry of Health, Community Development, Gender, Elderly and Children. [Internet]. National Plan for Reproductive, Maternal, Newborn, Child and Adolescent Health & Nutrition (2021/2022-2025/2026) One Plan III. Tanzania; 2021. [cited 2023 Sept 17]. Available from: <https://www.globalfinancingfacility.org/sites/gff_new/files/Tanzania-One-Plan-III.pdf>
28. Uganda: Ministry of Health. [Internet]. Reproductive, Maternal, Newborn, Child, Adolescent and Healthy Aging Sharpened Plan II 2022/23–2027/28. Uganda; 2022. [cited 2023 Sept 18]. Available from: <https://www.globalfinancingfacility.org/sites/gff_new/files/Uganda-GFF-Investment-Case-2022-2027.pdf>
29. Zambia: Republic of Zambia. [Internet]. Zambia RMNCAH-N Investment Case 2022-2024. Zambia; 2022. [cited 2023 Sept 19]. Available from: <https://www.globalfinancingfacility.org/sites/default/files/2023-03/Zambia-RMNCAH-N-Investment-Case.pdf>
30. Zimbabwe: Ministry of Health and Childcare, Republic of Zimbabwe. [Internet]. Investment Case for the National Health Strategy 2021-2025. Republic of Zimbabwe; 2020. [cited 2023 Sept 28]. Available from: <https://www.globalfinancingfacility.org/sites/gff_new/files/Zimbabwe-GFF-Investment-Case.pdf>
