# Supplementary material for: Financing of adolescent health and wellbeing in Bangladesh, Ethiopia and Nigeria: a case study of the Global Financing Facility
Source: BMC Glob Public Health. 2026 Sep 22;4:93. doi: 10.1186/s44263-026-00332-4 (PMC13599230; doi:10.1186/s44263-026-00332-4)
Supplement: Supplementary file 3 [file 44263_2026_332_MOESM3_ESM.docx]

**Financing of adolescent health and wellbeing in Bangladesh, Ethiopia and Nigeria: a case study of the Global Financing Facility**

**Supplementary Material 3: Topic guides (Bangladesh)**

**Guide for interviews with key-informants**

1. How have you heard of/been working with/collaborating with the GFF? Since when? *(introductory question – adjust depending on interviewee)*
2. Could you please describe the funding landscape in Bangladesh for adolescent health and well-being (AHW)?
   a. Where does most of the funding for AHW come from?
   b. How does this compare to what the national fundings landscape looks like for other areas, e.g., maternal health, child health, etc.?
   c. What are the main areas related to AHW that receive funding in Bangladesh?
   d. Are there any key areas of AHW that do not receive funding?
   e. *If not mentioned:* What about funding targeting mental health/education/violence/sexual reproductive health and rights?
3. How is AHW reflected in relevant documents in Bangladesh (e.g., policies, strategies, reports)?
   a. Do targets and indicators related to AHW exist? If yes, what are they?
   b. How are those targets and indicators monitored and evaluated?
4. What AHW program activities are being implemented in Bangladesh?
   a. What are these programs?
   b. Who are the main organizations implementing these programs?
   c. Where have these programs been implemented; in all regions?
   d. Are marginalized adolescents being reached, e.g., adolescents with disabilities, those living in rural areas, those who experienced child marriage, etc.?
   e. How do programs consider issues such as gender and age?
5. How would you describe the opportunities and challenges for investment in AHW?
   a. … in general?
   b. … for the GFF?
   c. In how far do the opportunities/challenges also apply to other areas or target groups, e.g., women and children?
6. Thinking more about the GFF funding for AHW...
   a. What do you think is working well?
   b. What do you identify as challenges?
   c. Do you think GFF funding has been catalytical, e.g., for getting additional funding for AHW? If yes, could you elaborate?
7. What do you perceive as the impact that the GFF funding has had on AHW in Bangladesh?
   a. What could be done to increase its impact in the future?
8. How can the GFF improve its collaboration to advance AHW with your organization/institution? *(Depending on the organizational/institutional affiliation of the interviewee, refer to collaboration with that particular organization/institution, e.g., government, technical partners, civil society organizations, etc.)*
9. How does the GFF invest in AHW in Bangladesh? *(this question is for specifically for interviewees from the GFF)*
   a. What was the process of country selection globally and the process of prioritization of AHW in the country?
   b. How does the investment in AHW in Bangladesh compare to the investment in women and children?
   c. What are the plans for the GFF in terms of AHW in Bangladesh?
10. What role have young people (under 25 years) played in the GFF process in Bangladesh?
    a. What role have young people played more generally in terms of improving AHW?
11. Do you have any other comments on the GFF and/or investments in AHW in Bangladesh?

**Guide for focus group discussions with adolescents**

1. When you think about your health and well-being, can you tell us about a positive experience?
   a. What would you say is going well with regards to your health and well-being?
   b. Have there been programs or policies that have supported these successes?
2. When you think about your health and well-being, can you tell us about a negative experience?
   a. What would you describe as difficulties you or your friends have faced regarding your health and well-being?
   b. Have there been any programs or policies that have helped you or your friends with these difficulties?
3. What would you describe as your main needs for being healthy and feeling well?
   a. In how far would you say that your needs have been met?
   b. What is needed to meet your needs?
4. Which services do you or your friends use to improve your health and well-being?
   a. *If not mentioned:* What about preventive/curative/public/private health services?
   b. In how far would you describe the health services you use as “youth friendly”?
   c. *If not mentioned:* Have you heard of any programs targeting mental health/education/violence/sexual reproductive health and rights?
   d. Which services have been most important to you or your friends?
   e. What do you or your friends like or dislike about those services?
   f. How have these services developed over the past few years?
5. Have you been engaged, e.g., as a youth representative, in any national or sub-national meetings about your health and well-being? If yes, could you elaborate?
6. Have you discussed your health and well-being with any government representatives? If yes, could you tell us more about that experience?
   a. Are there any other people you have discussed your health and well-being with?
   b. In how far do you feel supported and listened to by the government and other people with regards to your health and well-being?
7. Could you describe any activities you are involved in, which aim to improve your health and well-being?
   a. Who else is engaged in these activities?
   b. Who supports these activities? How?
   c. Do you know if these activities receive any funding, and if so from whom?
8. When you think of investments in your health and well-being more generally, e.g., through additional funding: Would you say that it is easy or difficult to invest in your health and well-being? Why?
9. Do you have any other comment related to your health and well-being and how it is or should be supported?

**Guide for focus group discussions with teachers or health professionals**

1. When you think about the health and well-being among your students/patients, can you tell us about a positive experience?
   a. What would you say is going well with regards to their health and well-being?
   b. Have there been programs or policies that have supported these successes?
2. When you think about the health and well-being among your students/patients, can you tell us about a negative experience?
   a. What would you describe as difficulties your students/patients have faced regarding their health and well-being?
   b. Have there been any programs or policies that have helped them with these difficulties?
3. What would you describe as your students’/patients’ main needs for being healthy and feeling well?
   a. In how far would you say that their needs have been met?
   b. What is needed to meet their needs?
4. Which services do your students/patients use to improve their health and well-being?
   a. *If not mentioned:* What about preventive/curative/public/private health services?
   b. In how far would you describe the health services as “youth friendly”?
   c. *If not mentioned:* Have you heard of any programs targeting adolescent mental health/education/violence/sexual reproductive health and rights?
   d. Which services have been most important to your students/patients?
   e. What do you or your students/patients like or dislike about those services?
   f. How have these services developed over the past few years?
5. Have your or your students/patients been engaged in any national or sub-national meetings about their health and well-being? If yes, could you elaborate?
6. Have you or your students/patients discussed their health and well-being with any government representatives? If yes, could you tell us more about that experience?
   a. Are there any other people you or your students/patients have discussed their health and well-being with?
   b. In how far do you or your students/patients feel supported and listened to by the government and other people with regards to their health and well-being?
7. Could you describe any activities you or your students/patients are involved in, which aim to improve your health and well-being?
   a. Who else is engaged in these activities?
   b. Who supports these activities? How?
   c. Do you know if these activities receive any funding, and if so from whom?
8. When you think of investments in your students’/patients’ health and well-being more generally, e.g., through additional funding: Would you say that it is easy or difficult to invest in their health and well-being? Why?
9. Do you have any other comment related to your students’/patients’ health and well-being and how it is or should be supported?
